# Genetic and non-genetic determinants of vitamin D status: a polygenic score analysis in elite athletes

**DOI:** 10.3389/fgene.2026.1838157

**Published:** 2026-05-28

**Authors:** Sebastian Hacker, Lukas Reichert, Claudia Lenz, Sabrina Henne, Friederike David, Stefanie Heilmann-Heimbach, Karen Zentgraf, Karsten Krüger

**Affiliations:** 1 Department of Exercise Physiology and Sports Therapy, Institute of Sports Science, Justus Liebig University Giessen, Giessen, Germany; 2 Institute of Sports Science, Work Unit Movement and Exercise Science, Goethe University Frankfurt, Frankfurt am Main, Germany; 3 Institute of Human Genetics, School of Medicine & University Hospital Bonn, University of Bonn, Bonn, Germany

**Keywords:** elite athletes, gene-environment interaction, genetics, polygenic score, seasonality, supplementation, vitamin D

## Abstract

**Introduction:**

Vitamin D plays a critical role in bone metabolism, immune regulation, and muscle performance. Serum 25-hydroxyvitamin D [25(OH)D] levels are shaped by both genetic and non-genetic determinants. To explore their combined role, we investigated the association between a polygenic score (PGS) for vitamin D metabolism and serum 25(OH)D concentrations in elite athletes.

**Methods:**

Serum vitamin D status was measured in 473 German national squad athletes. PRSice-2 was used for PGS development. Linear regression models compared the predictive utility of an overall model (PGS plus covariates) versus a null model (covariates only). Covariates included age, sex, competition environment, ambient UVB dose, and supplementation.

**Results:**

Serum 25(OH)D was significantly predicted by the standardized PGS (β = 4.04), age (β = 8.59), supplementation (β = 20.86), and cumulative weighted UVB dose (cw-D-UVB, β = 7.18; all p < 0.05). The full model explained 31.7% (12.1%) of variance (adjusted R2 = 0.235), with the PGS contributing an incremental 1% (1%). Model performance yielded a mean absolute error of 18.64 nmol/L (3.18 nmol/L) and root mean squared error of 25.27 nmol/L (4.24 nmol/L). No significant interactions were found for PGS × cw- D-UVB, PGS × supplementation status and PGS × competition environment.

**Discussion:**

The findings indicate that genetic determinants account for a small proportion of the total variance in vitamin D status. Stronger predictors are non-genetic determinants such as supplementation, UVB exposure, and age, highlighting the predominant role of environmental influences. Given the limited explanatory power of the PGS, individualized management strategies should primarily focus on modifiable factors such as sun exposure and supplementation.

## 1 Introduction

Vitamin D, a secosteroid hormone with pleiotropic effects, is essential for skeletal integrity, immune competence, and neuromuscular function. Its circulating levels are determined by both environmental inputs most notably ultraviolet B (UVB) radiation, dietary supplementation, and by inherited genetic variation (Revez et al., 2020; Shraim et al., 2025). In athletic populations, insufficient vitamin D status has been linked to elevated risk of stress fractures, respiratory tract infections, and muscle injuries (Crescioli, 2022; de la Puente Yagüe et al., 2020). Maintaining adequate vitamin D status may thus be critical for elite athletes, as insufficient vitamin D levels could contribute to greater time lost from training or competition due to illness or injury (Bauer et al., 2018; Harju et al., 2022).

In recent years, genome-wide association studies (GWAS) have helped identifying the genetic architecture of complex traits (Revez et al., 2020; Visscher et al., 2017). Specifically, GWAS of serum 25-hydroxyvitamin D [25(OH)D] levels have pinpointed several loci associated with vitamin D conversion, transport, and bioavailability, while also enabling the estimation of the individual effect sizes of these genetic variants (Revez et al., 2020). This knowledge allows for the construction of polygenic scores (PGSs), which quantify an individual’s genetic predisposition to a certain trait (Wand et al., 2021). Typically, a polygenic score (PGS) is calculated by weighing an individuals’ sum of risk alleles with the respective estimated effect sizes from a GWAS on the phenotype of interest (Choi et al., 2020; Wand et al., 2021). Earlier studies have employed PGSs to estimate the genetic contribution to vitamin D status across various clinical and general populations, including cohorts with osteoporosis, multiple sclerosis, or cardiometabolic risk (Jiang et al., 2018; Manousaki et al., 2020). While these studies consistently identify statistically significant associations, PGSs typically account for only a small proportion of the variance in serum 25(OH)D concentrations. Moreover, most underlying GWAS have been conducted in older, non-athletic populations with relatively uniform environmental exposures (Jiang et al., 2018; Revez et al., 2020), limiting the ecological validity of their findings for elite athletes.

Athletic populations are exposed to distinct environmental and behavioral factors such as high training loads, variable sunlight exposure, individualized nutrition, and supplementation practices that may interact with genetic predisposition in shaping vitamin D levels (Ammar et al., 2023; Fernández-Lázaro et al., 2025; Usategui-Martín et al., 2022). Previous studies reported that a substantial proportion of athletes were classified as having insufficient or deficient serum 25(OH)D levels (<75 nmol/L), underscoring the relevance of this issue in high-performance settings (Farrokhyar et al., 2015; Hacker et al., 2025). Despite the physiological relevance of vitamin D for this population, genetic investigations in these cohorts remain scarce. In a previous study we demonstrated that season and competition environment significantly influence 25(OH)D levels in elite athletes and incorporated candidate SNP analysis to explore genotype-phenotype relationships (Hacker et al., 2025). Yet, no study has systematically examined the combined contribution of polygenic predisposition and non-genetic modifiers in an elite sports context. Addressing this gap requires integrated, context-specific models that capture how genetic predisposition, and non-genetic factors jointly contribute to vitamin D status in athletic populations.

An integrated model combining genetic, demographic, and environmental variables can help elucidate underlying interactions (Wand et al., 2021). Recent advances in sport and performance research emphasize such a multifactorial gene–environment interaction model, in which genetic predispositions dynamically interact with environmental and behavioral influences to shape phenotypic outcomes (Ullén et al., 2016; Zentgraf and Raab, 2023). Within this model, genetic variants related to vitamin D metabolism, transport, or receptor function may exert differing effects depending on environmental conditions such as sunlight exposure, dietary vitamin D intake, supplementation practices, geographical latitude, or seasonal variation. For instance, an athlete genetically predisposed to lower serum 25(OH)D levels might compensate effectively through higher sun exposure or tailored nutritional/supplementation interventions, thus mitigating potential negative impacts on health and performance (Rojano-Ortega and Berral-de la Rosa, 2023; Wyatt et al., 2024). Therefore, integrative models that incorporate both genetic and non-genetic determinants could enhance the explanatory and predictive power regarding serum 25(OH)D levels, enabling more personalized and precise recommendations tailored to individual athletes. Such multifactorial approaches acknowledge the complexity of athletic performance, emphasizing that optimal vitamin D status–and by extension, injury prevention, immune function, and muscle performance–is not determined solely by genetics or environment alone, but rather via their nuanced interactions.

To investigate the combined role of genetic and non-genetic determinants, we investigated the extent to which a PGS derived from an established genome-wide association study predicts serum 25(OH)D concentrations in German national squad athletes. By incorporating both genetic and non-genetic determinants into a multivariable model, we aimed to (1) quantify the relative contribution of genetic predisposition to interindividual differences in vitamin D levels, and (2) examine how genetic and non-genetic determinants jointly contribute to vitamin D status in high-performance settings, potentially informing personalized strategies for optimizing vitamin D status. We hypothesized that the PGS would significantly predict serum 25(OH)D levels, but that its effect would be modulated by non-genetic variables such as supplementation, UVB exposure, and competition environment.

## 2 Methods

This cross-sectional study is part of the “in:prove” project (Individualized performance development in elite sport through holistic and transdisciplinary process optimization) funded by the German Federal Institute of Sport Science (grant number 081901/21–25). The study protocol was approved by the institutional ethics committee of the University of Giessen, Germany (date of approval: 2022–05-10, approval number: AZ 55/22) and adhered to the Declaration of Helsinki for human research. Prior to participating in the study all athletes received detailed written and verbal information and gave their written informed consent.

In total, we analyzed data of 473 German elite athletes aged 13–35 years (mean age 18.7 (4.3) years) See Table 1 for participant characteristics. Their mean serum 25(OH)D concentration was 71.73 (31.2) nmol/L, ranging from 13.75 to 250 nmol/L. The athletes represented 10 sports disciplines: 3 × 3 basketball, artistic gymnastics, bobsleigh, ice hockey, modern pentathlon, rhythmic gymnastics, skeleton, table tennis, trampoline gymnastics, and volleyball. At the time of measurement, all athletes were part of the German national (junior) squad.

**TABLE 1:** Participant characteristics.

Variable	*n*	%
Total participants	473	100
Sex		
*Male*	259	55
*Female*	214	45
Competition environment		
*Indoor*	363	77
*Outdoor*	110	23
Supplementation		
*No*	181	38
*Yes*	47	10
*Unknown*	245	52
Month of measurement		
*January*	28	6
*February*	0	0
*March*	8	2
*April*	50	11
*May*	81	17
*June*	88	19
*July*	76	16
*August*	21	4
*September*	20	4
*October*	44	9
*November*	54	11
*December*	3	1

### 2.1 Blood sampling

Venous blood was drawn from the median cubital vein of the forearm for both serum 25(OH)D analysis and genotyping. In detail, two samples were obtained using the following tubes: 7.5 mL Serum Gel and 7.5 mL EDTA (Sarstedt, Nümbrecht, Germany). Serum 25(OH)D concentrations were determined by an accredited medical laboratory (SYNLAB Medizinisches Versorgungszentrum, Bad Nauheim, Germany) using a Chemiluminescent Microparticle Immunoassay (CMIA). The analytical detection range for the assay was 8.75–385.5 nmol/L. Prior to genotyping, the 7.5 mL EDTA tube was centrifuged at 2000 rpm for 10 min at room temperature to separate plasma from cellular components. Following centrifugation, the EDTA plasma was carefully collected, aliquoted, and stored at −80 °C for future analyses unrelated to the present study, while the remaining cellular fraction was likewise stored at −80 °C until DNA extraction and genotyping. All samples were processed and stored in this manner.

### 2.2 DNA extraction, genotyping and imputation

Genomic DNA was extracted from whole blood using the Chemagic Magnetic Separation Module I system (Perkin Elmer Chemagen Technology Inc., Baesweiler, Germany). Genotyping was performed with the Illumina Global Screening Array (GSAv3.0) including Medical Disease and Psych modules (MD + Psych; Illumina Inc., San Diego, CA, United States) at Life and Brain GmbH, Bonn, Germany. All laboratory procedures followed the manufacturer’s standard protocols.

Genotype data were filtered in PLINK1.9 (Chang et al., 2015; Purcell and Chang, 2024) for MAF ≥1%, and call rate ≥98%. Autosomal heterozygosity, X-chromosomal heterozygosity, genetic relatedness and principal components (PCs) were calculated based on pruned variants (r^2^ ≤ 0.2) with MAF ≥5% and Hardy-Weinberg equilibrium (HWE) exact test *p*-value (*p* > 10^−3^). Ancestry was inferred using KING (version 2.3.2) using 1000 Genomes Project data as a reference. Samples were excluded based on deviating autosomal heterozygosity (|Fhet| > 0.2), mismatching reported and inferred sex and inferred non-European ancestry. Additionally, genetic outliers (>6 standard deviations from the mean in PC1 – PC10) were iteratively excluded.

The data were phased using EAGLE (version 2.4.1) and 1,000 Genomes Phase 3 haplotypes, followed by imputation to the 1,000 Genomes Phase 3 reference panel using Minimac4 (version 4.1.6).

In total, 552 samples entered quality control, and 499 samples remained. However, at the time of analysis we did not have data regarding serum 25(OH)D levels for 26 athletes, which led to the final sample size of 473 athletes.

### 2.3 Ambient UVB dose

To estimate individual ambient UVB exposure, daily vitamin D-effective UVB dose data (D-UVB, kJ/m^2^) were obtained from the Tropospheric Emission Monitoring Internet Service (TEMIS, Copyright © KNMI/ESA, https://www.temis.nl/) database (Van Geffen et al., 2017). The D-UVB values are derived from a parameterization incorporating satellite-based ozone observations, the solar zenith angle, and the vitamin D action spectrum, with corrections applied for surface elevation, surface albedo, sun-earth distance, and cloud-cover (Zempila et al., 2017). An individual cumulative and weighted ambient D-UVB value (cw-D-UVB) was subsequently calculated for each athlete using the R package ‘UVdose’ (Shraim and Zgaga, 2025), following the approach described in O’Sullivan et al. (2017). Briefly, daily D-UVB doses were retrieved over a 135-day period preceding the date of blood collection. Because this window was defined individually relative to each athlete’s blood collection date, seasonal variation in UVB availability was inherently reflected in the cw-D-UVB estimate, and no additional seasonal adjustment was applied. As individual residential addresses were unavailable, a single central German reference location (51.22°N, 9.36°E) was applied uniformly across all athletes as a pragmatic approximation. To account for the ongoing physiological utilization of vitamin D, daily values were exponentially weighted according to the 35-day half-life of the UVB-vitamin D effect, such that more temporally distant UVB exposure contributes progressively less to the cw-D-UVB estimate.

### 2.4 Polygenic score development

Prior to PGS calculation, we applied the following quality control procedures to our target dataset of elite athletes. Exclusion criteria were: variants with a MAF <1%, call rate <95%, HWE *p*-value of <10^−6^ and imputation *R* ^2^ ≥ 0.3. A detailed description regarding the quality control procedures of the base GWAS can be found in Revez et al. (2020).

PGSs for serum 25(OH)D concentration were calculated as described in Choi et al. In brief, we used the software PRSice-2 (version 2.3.5) and a clumping-thresholding approach (Choi and O’Reilly, 2019) to calculate a series of PGSs with *p*-value thresholds from 5 × 10^−8^ up to 0.5 based on publicly available summary statistics from a GWAS of 25(OH)D by Revez et al. (N = 417,580 Europeans; PGS Catalog: PGP000228) (base GWAS) (Revez et al., 2020). The generated PGSs were subsequently applied to our target dataset of elite athletes. The optimal *p*-value threshold for the PGS generation was defined as the one explaining the highest proportion of variance in serum 25(OH)D levels in the target population, adjusted for age, sex, cw-D-UVB, competition environment (indoor/outdoor), supplementation status (yes, no, unknown), and the first three principal components of genetic ancestry. These covariates were chosen to resemble the ones implemented in the base GWAS by Revez et al. (2020). The disciplines artistic gymnastics, ice hockey, rhythmic gymnastics, table tennis, trampoline gymnastics, and volleyball were categorized as indoor disciplines, while 3 × 3 basketball, bobsleigh, modern pentathlon, and skeleton were categorized as outdoor disciplines. Dietary intake was assessed using a 3-day nutrition protocol, in which athletes documented all foods, beverages, and supplements consumed, including quantities. Protocols were analyzed using DGExpert (version 2.0.37; German Nutrition Society), with data subjected to a quality check prior to analysis. If multiple protocols were submitted, only the first was included in the analysis. Further methodological details are described elsewhere (Hacker et al., 2025).

### 2.5 Statistical analyses

Statistical analyses were performed in R version 4.5.2 (R Core Team, 2025) using Positron version 2026.03.0 (Posit Software, 2026). Figures were created using the R package ‘ggplot2’ (Wickham, 2016). Descriptive statistics are reported as mean (SD) unless stated otherwise. All continuous predictor variables were z-standardized (mean = 0, SD = 1) prior to analyses.

To assess the relative contribution of genetic and non-genetic predictors to serum 25(OH)D concentrations, we fitted two multiple linear regression models: a full model including the standardized PGS and all covariates, and a null model including only the covariates. Model performance was evaluated using 5-fold cross-validation, estimating *R* ^*2*^, adjusted *R* ^*2*^, mean absolute error, and root mean squared error. The explanatory power of the PGS is reported as the incremental *R* ^*2*^, i.e., the increase in *R* ^*2*^ when the PGS was added to the null model (Choi et al., 2020). Model assumptions (homoscedasticity, normality of residuals, and multicollinearity) were assessed with the ‘performance’ package (Lüdecke et al., 2021). In cases of assumption violations, robust regression with heteroscedasticity-consistent standard errors (HC4) was applied.

To examine whether PGS values differed across groups defined by vitamin D status, we conducted a one-way analysis of variance (ANOVA). Vitamin D status was categorized according to previously suggested thresholds: sufficient (≥75 nmol/L), insufficient (50–75 nmol/L), and deficient (<50 nmol/L) (Holick et al., 2011). Post-hoc comparisons were performed using Tukey’s HSD. Statistical significance was set at *p* < 0.05 (two-tailed), and effect sizes with 95% confidence intervals are reported where applicable.

To exploratively assess whether the association between the PGS and serum 25(OH)D concentrations was moderated by environmental or behavioral factors, three additional interaction models were fitted. Building on the full regression model, we separately introduced the product terms PGS × cw-D-UVB, PGS × supplementation status and PGS × competition environment. All interactions were specified based on theoretical considerations regarding gene-environment interplay in vitamin D metabolism: ambient UVB radiation, supplementation and outdoor discipline were hypothesized to modulate the genetic effect by providing exogenous sources of vitamin D. Model fit was evaluated by comparing each interaction model against the full model using robust Wald tests, with heteroscedasticity-consistent standard errors (HC4) applied consistently across all interaction models. Given the exploratory nature of these analyses, results are interpreted as hypothesis-generating and no adjustments for multiple comparisons were applied.

## 3 Results

### 3.1 Polygenic score performance and analysis of non-genetic determinants

**FIGURE 1:**
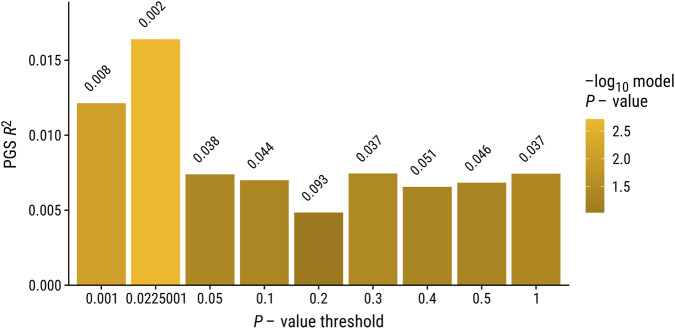
Polygenic score (PGS) performance for serum 25-hydroxyvitamin D [25(OH)D] across varying GWAS p-value thresholds. The plot shows the proportion of variance in serum 25(OH)D concentrations explained (PGS R2) by scores constructed using SNPs selected at varying GWAS p-value thresholds (0.001 to 1). Bars are colored by -log10-transformed model p-values, with lighter colors indicating greater statistical significance. Annotated values above each bar represent the corresponding model p-values.

The highest predictive performance was observed at a *p*-value threshold of 0.0225,001. At this threshold, the PGS included 18,393 SNPs and explained 1.6% of the variance in serum 25(OH)D concentration (*R* ^2^ = 0.016). PGS performance across other selected GWAS *p*-value thresholds is shown in Figure 1.

**FIGURE 2:**
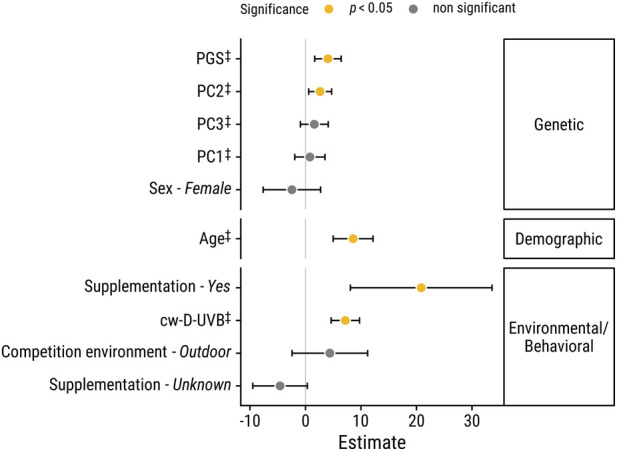
Forest plot of predictors included in the multiple linear regression model. Variables are grouped by category (Genetic, Demographic, Environmental/Behavioral) and ordered within each category by the magnitude of their estimated effects. Dots indicate regression coefficients; horizontal lines denote 95% confidence intervals. Numeric variables marked with ‡ were standardized prior to analysis. Color indicates significance: yellow (p < 0.05), gray (not significant); color is for visual emphasis only and not essential for interpretation. PGS, polygenic score; PC, principal component.

**FIGURE 3:**
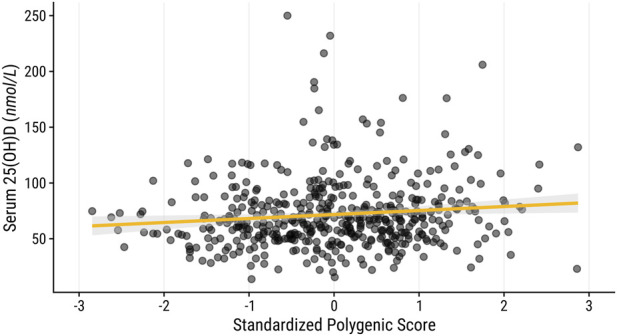
Association between the standardized polygenic score and serum 25-hydroxyvitamin D [25(OH)D levels (in nmol/L).

The full model was significant, *F* (10, 462) = 13.06, *p* < 0.001, and explained 22% of the variance in serum 25(OH)D levels (*R* ^2^ = 0.22, adjusted *R* ^2^ = 0.204). The model intercept was β = 72.12, 95% CI [67.72, 76.51], *p* < 0.001. The PGS (β = 4.04, 95% CI [1.65, 6.44], *p* < 0.001), age (β = 8.59, 95% CI [4.99, 12.19], *p* < 0.001), supplementation–*yes* (β = 20.86, 95% CI [8.06, 33.65], *p* = 0.001), the cw-D-UVB (β = 7.18, 95% CI [4.62, 9.74], *p* < 0.001) and the second principal component (PC2; β = 2.64, 95% CI [0.57, 4.72], *p* = 0.012) were significant predictors of serum 25(OH)D levels (Figure 2). The model was estimated using robust regression with heteroscedasticity-consistent standard errors (HC). The small positive association between serum 25(OH)D and PGS is shown in Figure 3. See Supplementary Table S1 for the regression table.

### 3.2 Validation of polygenic score performance

On average the full model explained 31.7% (12.1%) of the variance in serum 25(OH)D concentration, the null model explained 30.9% (11.5%), and the incremental *R* ^2^ was 1% (1%) (range: 0%–2.1%). The average mean absolute error of the full model was 18.64 nmol/L (3.18 nmol/L), and the root mean squared error was 25.27 nmol/L (4.24 nmol/L). All models were estimated using robust regression with heteroscedasticity-consistent standard errors (HC). Detailed information on PGS performance per fold is displayed in Supplementary Table S2.

### 3.3 Polygenic score and medical reference values

A one-way ANOVA was conducted to examine whether the PGS differed between vitamin D status groups defined by previously suggested thresholds. The categories were: deficient <50 nmol/L, insufficient 50–75 nmol/L, sufficient ≥75 nmol/L (Holick et al., 2011).

**FIGURE 4:**
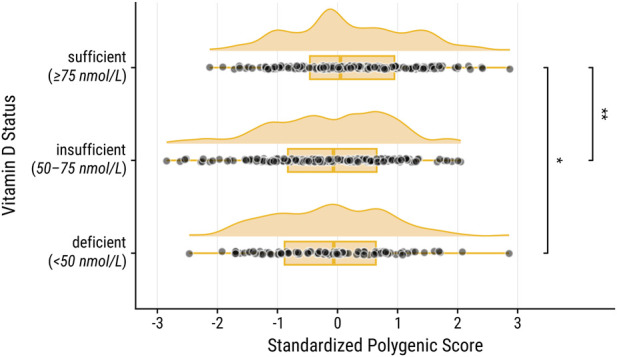
Raincloud plots of the standardized polygenic risk score distributions stratified by serum 25-hydroxyvitamin D status. Color is used solely for visual emphasis and is not required for interpretation. Asterisks denote significance levels: * indicates p < 0.05 and ** indicates p < 0.01.

The analysis revealed a statistically significant effect of vitamin D status on PGS, *F* (2, 470) = 5.36, *p* = 0.005, indicating differences between groups. Tukey’s HSD *post hoc* comparisons showed that participants with sufficient vitamin D status had a higher PGS compared to both the deficient (mean difference = 0.31, 95% CI [0.02, 0.61], *p* = 0.035) and the insufficient group (mean difference = 0.31, 95% CI [0.07, 0.55], *p* = 0.008) (Figure 4). No statistically significant difference was found between the deficient and insufficient groups (*p* = 1).

### 3.4 Exploratory interaction analyses

The PGS × cw-D-UVB interaction was non-significant (β = 1.18, 95% CI [-1.31, 3.67], *p* = 0.351; Wald F (1, 461) = 0.872, *p* = 0.351), as was the PGS × competition environment interaction (β = 2.33, 95% CI [-4.22, 8.88], *p* = 0.485; Wald F (1, 461) = 0.488, *p* = 0.485), and the PGS × supplementation interaction (β_yes_ = 9.81, 95% CI [-1.03, 20.65], *p* = 0.076, β_unknown_ = 2.04, 95% CI [-2.7, 6.79], *p* = 0.398; Wald F (2, 460) = 1.692, *p* = 0.185). None of the interaction models provided a significantly better fit than the full model.

## 4 Discussion

To the best of our knowledge, this study is among the first to evaluate the combined influence of polygenic predisposition and non-genetic factors on serum 25(OH)D concentrations in an elite athletic population. Our findings reveal that while a PGS for vitamin D metabolism predicts individual variability, its explanatory power remains modest compared to non-genetic factors such as supplementation, UVB exposure (seasonality), and age.

The small albeit statistically significant contribution of the PGS to serum 25(OH)D levels aligns with findings from previous population-based cohorts, where genetic predictors account for only a small fraction of the variance in 25(OH)D (Manousaki et al., 2020; Revez et al., 2020). For instance, Manousaki et al. reported that 138 genome-wide significant SNPs explained 4.9% of the variance in 25(OH)D levels, with common variants accounting for 3.1% and low-frequency and rare variants for 1.8% (Manousaki et al., 2020). When estimating heritability from all SNPs independent of GWAS *p*-value the explained proportion of variance was 16.1% (Manousaki et al., 2020). Similarly, Revez and colleagues estimated the SNP-based heritability at 13% (Revez et al., 2020). In contrast, twin studies reported highly variable estimates of the heritability of serum 25(OH)D concentrations, ranging from 0% to 86% (Jiang et al., 2019). Collectively, these findings highlight that while genetic architecture contributes to vitamin D status, modifiable non-genetic factors remain dominant drivers, particularly in athletic populations where supplementation strategies and seasonal training environments are central to performance and health management.

There has been growing interest in leveraging genetic information to refine vitamin D research, particularly through the development of polygenic scores. A vitamin D specific PGS could enable the identification of individuals genetically predisposed to insufficient 25(OH)D levels and thus inform tailored nutritional and supplementation strategies. Such approaches hold promise for advancing precision nutrition in both clinical and performance contexts. However, most prior studies have been restricted to the analysis of individual SNPs or variants surpassing conventional genome-wide significance thresholds, thereby potentially overlooking the broader polygenic architecture underlying vitamin D metabolism (Chandler et al., 2018; Engelman et al., 2013; Fernández-Lázaro et al., 2025; Gabdulkayum et al., 2026; Krasniqi et al., 2025; Nissen et al., 2014; Shao et al., 2018; Vasileiou et al., 2023; Zhang et al., 2013). In a previous study, Hatchell and colleagues evaluated the predictive utility of two ancestry-matched vitamin D PGSs in older adults of African and European descent (Hatchell et al., 2019). Among participants with European ancestry, the optimal score comprising 341 SNPs accounted for 1.4% of the variance in log-transformed 25(OH)D levels in the target dataset (*n* = 1,000; *p* = 9.3 × 10^−5^) and 1% of the variance in the validation cohort (*n* = 8,569; *p* = 1.1 × 10^−23^) (Hatchell et al., 2019). In comparison, the vitamin D PGS derived in the present study explained 1.6% of the variance in serum 25(OH)D in the target dataset of elite athletes; however, subsequent 5-fold cross-validation yielded a more conservative estimate of approximately 1% (1%). While these values indicate a modest predictive power of current PGSs, they align with previous findings and highlight both the polygenic nature of vitamin D regulation and the need for larger, ancestry-diverse datasets to improve prediction accuracy. Furthermore, Hatchell and colleagues demonstrated that individuals in the highest genetic risk decile had, on average, 7–7.5 nmol/L lower serum 25(OH)D concentrations compared to those in the lowest decile, with significant effects observed in participants of European ancestry (*p* = 3.2 × 10^−13^) and in those of African ancestry (*p* = 0.046) (Hatchell et al., 2019). Similarly, in our model serum 25(OH)D concentrations increased with higher PGS values. Athletes with a PGS one standard deviation above the mean had, on average, 4.04 nmol/L higher serum 25(OH)D compared to those at the mean. Moreover, considering commonly applied medical thresholds, athletes with sufficient vitamin D levels, i.e., serum 25(OH)D ≥ 75 nmol/L, showed a higher PGS compared to athletes with insufficient (50–75 nmol/L) or deficient (<50 nmol/L) status. These findings are consistent with the notion that genetic predisposition influences vitamin D metabolism and circulating concentrations, potentially through variants affecting synthesis, transport, or degradation pathways (Manousaki et al., 2020; Revez et al., 2020).

As mentioned earlier genetic predispositions dynamically interact with environmental and behavioral influences to shape phenotypic outcomes (Ullén et al., 2016; Zentgraf and Raab, 2023). In a recent study, Hatchell et al. examined the interaction of an ancestry-matched PGS with both ambient UV radiation and vitamin D intake (Hatchell et al., 2020). In participants with European ancestry, they found a statistically significant interaction between PGS and UV radiation (β = 0.017, *p* = 0.021), indicating that individuals with a higher PGS benefited more from available UV radiation in terms of 25(OH)D levels. In contrast, the interaction between PGS and vitamin D intake was not significant (β = 0.0006, *p* = 0.74), suggesting that the effect of dietary vitamin D on 25(OH)D levels was similar regardless of genetic risk. Nonetheless, in those who met the Institute of Medicine vitamin D intake guidelines (600 IU/day for those 1–70 years old and 800 IU/day for those over 70) (National Institutes of Health, 2025), the proportion achieving adequate 25(OH)D levels (≥50 nmol/L) increased with higher PGS ranging from 71.7% in the lowest quartile to 89% in the highest (*p* = 0.018) (Hatchell et al., 2020). However, our analysis revealed no interaction between PGS and cw-D-UVB, suggesting that the genetic effect on serum 25(OH)D does not meaningfully vary as a function of ambient UVB exposure. Notably, both the main effect of the PGS and cw-D-UVB were independently associated with serum 25(OH)D, indicating that genetic predisposition and seasonal UVB availability contribute additively, rather than interactively, to vitamin D status. Compared to Hatchell et al., these differences may be attributable to variations in PGS calculation (e.g., the use of different GWAS summary statistics) and differences in the estimation of prior UV exposure (Hatchell et al., 2020). Across all three interaction models, the pattern of main effects remained largely consistent with the full model. Taken together, these exploratory interaction analyses suggest that genetic predisposition and environmental or behavioral factors contribute largely independently to vitamin D status in this athletic sample.

In addition to these findings, we observed that age was positively associated with serum 25(OH)D. In our model, a one standard deviation increase in age corresponded to an estimated increase of 8.59 nmol/L in serum 25(OH)D. This association may reflect greater nutritional awareness and stronger adherence to performance-oriented dietary strategies among older athletes with longer involvment in professional sports, potentially leading to more consistent vitamin D intake and sun exposure practices (Krzywanski et al., 2016). Accordingly, these findings highlight the potential value of earlier and more comprehensive nutrional education and support, especially in junior level athletes. Moreover, in line with previous studies on vitamin D supplementation (Wyatt et al., 2024), our results suggest a potential benefit of personalized supplementation strategies for achieving higher serum 25(OH)D concentrations. Specifically, athletes reporting vitamin D supplementation had higher (β = 20.86) serum 25(OH)D levels compared to those not reporting supplementation. However, as over 50% of athletes did not report on their supplementation behavior and those who did rarely provided sufficient detail, no information regarding dose, duration or formulation was integrated into the statistical models, which may partly explain the wide confidence interval observed for this estimate (95% CI [8.06, 33.65]). Beyond supplementation, dietary vitamin D intake was not included in the present study. This may be particularly relevant in the context of elite sport, where structured nutritional programs supervised by sports nutritionists may include vitamin D-rich foods such as fatty fish, eggs, and fortified dairy products. The absence of dietary intake data could potentially lead to an overestimation of the relative contribution of UVB exposure, an underestimation of non-genetic determinants more broadly, and may have introduced residual confounding into the regression model. Previous studies indicate that, under typical conditions, dietary intake accounts for approximately 10%–20% of vitamin D status, whereas 80%–90% is derived from cutaneous synthesis induced by UVB radiation (Holick, 2004; Owens et al., 2018). Nonetheless, future studies should endeavor to incorporate comprehensive nutritional assessments to more accurately capture total vitamin D intake.

It is well documented that vitamin D levels are strongly influenced by seasonal variation (Holick et al., 2011; Rabenberg and Mensink, 2016). Accordingly, an individual cumulative and weighted ambient UVB exposure (cw-D-UVB) value was calculated for each athlete, incorporating daily ambient UVB doses at wavelengths capable of inducing cutaneous vitamin D synthesis over a 135-day period preceding blood collection. By using date-specific daily UVB data for each individual time window, this approach inherently captures seasonal differences in UVB availability, ensuring that variations in sunlight exposure prior to sampling are appropriately reflected in the cw-D-UVB estimates. This approach provides a more physiologically relevant approximation of recent UVB exposure compared to a single timepoint or unweighted measures, as it accounts for both temporal dynamics and biologically effective radiation. Consistent with this rationale, higher cw-D-UVB exposure was statistically significantly associated with higher serum 25(OH)D concentrations, indicating that athletes with greater ambient UVB exposure exhibited correspondingly higher vitamin D levels. This finding is likely attributable to the absence of sufficient UVB radiation for cutaneous vitamin D synthesis in Germany (Latitude 47–55°N) during the fall and winter months (Rabenberg and Mensink, 2016; Webb and Holick, 1988), resulting in pronounced seasonal fluctuations in vitamin D status.

Taken together, our findings suggest that PGSs may contribute modestly to the prediction of vitamin D inadequacy in elite athletes, while also highlighting the dominant role of modifiable non-genetic determinants. However, given that the explanatory power of the current vitamin D PGS was limited to approximately 1% of variance, its standalone clinical utility remains insufficient at this stage. Rather than serving as an independent predictive tool, PGS information may be most valuable when integrated with data on supplementation, age, and UVB exposure to inform more individualized strategies to optimize serum 25(OH)D concentrations. Notably, prior work has suggested that genetic predisposition may shape the responsiveness to supplementation (Didriksen et al., 2013; Mazahery and Von Hurst, 2015; Nimitphong et al., 2013; Usategui-Martín et al., 2022), pointing toward a gene–environment interplay with relevance for both preventive healthcare and performance management. In this regard, PGS-guided approaches may hold exploratory promise for identifying athletes at potentially higher risk of insufficiency, though larger studies with more comprehensive genomic data will be necessary before such approaches can meaningfully advance precision nutrition in sports.

### 4.1 Strength and limitations

A key strength of the present study is the joint modeling of genetic and non-genetic factors, allowing assessment of their combined contribution to vitamin D status. This integrative approach allows for a more nuanced understanding of the determinants of serum 25(OH)D concentrations in elite athletes. Another notable strength is the recruitment of a comparatively large cohort of elite athletes. In elite sports research, access to athletes is typically highly restricted, and studies are often limited by small sample sizes. The inclusion of such a substantial number of participants therefore not only enhances the robustness and statistical power of the analyses but also increases the generalizability and relevance of the findings for this population.

Nevertheless, some limitations should be acknowledged. First, the calculated PGS depends on the underlying GWAS summary statistics. Differences in variant availability between the base and target datasets may result in the loss of genetic information prior to score calculation. Second, regarding non-genetic determinants, the use of nutrition protocols is prone to bias, particularly underreporting, which may have affected the accuracy of information regarding the supplementation, including dose, duration, mode of administration, and formulation (vitamin D2 vs. D3). Third, although ambient UVB exposure preceding the date of blood collection was estimated, individual sun exposure behavior was not assessed. Factors such as sunscreen use, clothing habits, time spent outdoors, and travel to sunnier regions may substantially attenuate or enhance the actual cutaneous UVB dose received, irrespective of ambient UVB availability. This applies also to the dichotomization of competition environment into indoor and outdoor sports, as outdoor disciplines may differ considerably regarding training and competition schedules (e.g., time of day and corresponding solar intensity). Furthermore, the absence of individual residential data necessitated the use of a single central German reference location as a pragmatic approximation, which may not fully reflect regional variation in UVB exposure.

### 4.2 Future directions

Future research may utilize the findings of this study to investigate the inter-individual response to vitamin D supplementation in a longitudinal design. Incorporating both genetic and non-genetic (demographic/environmental/behavioral) factors may allow for a more precise supplementation strategy. Further, future studies need to address more diverse ethnicities as the main proportion of current research was conducted in populations with European ancestry (Moreno-Grau et al., 2024). In example, the PGS performance decays in populations when the best PGS model for Europeans is applied in, for example, South Asians, East Asians or Africans (Moreno-Grau et al., 2024). This hinders the effective use of PGSs in these populations and potentially increases health disparities (Moreno-Grau et al., 2024).

### 4.3 Conclusion

In conclusion, by integrating genetic and non-genetic determinants into a combined model, our study emphasizes the multifactorial nature of vitamin D regulation in high-performance settings and provides a preliminary framework for more individualized monitoring and targeted interventions in elite sport.

From a practical perspective, these findings suggest that maintaining adequate vitamin D intake, potentially supported by individualized supplementation strategies, may be particularly relevant during periods of limited UVB availability. In addition, structured nutritional education, especially at the junior athlete level, could contribute to the early establishment of appropriate dietary and sun exposure practices. Consideration of safe and sufficient UVB exposure, where feasible, should be balanced with established skin cancer prevention recommendations. Given the inter-individual variability observed, regular screening of serum 25(OH)D concentrations appears warranted to enable timely identification and correction of inadequacy. Future studies should additionally incorporate dietary vitamin D intake assessments to allow for a more complete characterization of its determinants. This is particularly relevant in light of the pleiotropic effects of vitamin D on physiological systems relevant to athletic health and performance, including musculoskeletal function, immune regulation, and recovery processes (de la Puente Yagüe et al., 2020; Ribbans et al., 2021).

## Data Availability

The datasets generated and/or analyzed during the current study are not fully publicly available due to ethical and legal restrictions, specifically concerning the raw genotype data of the participating athletes. A minimal dataset sufficient to reproduce the main findings of this study is publicly available at https://github.com/leistungsphysiologie/hacker-2026-frontiers. Additional data may be available from the corresponding author upon reasonable request and subject to ethical approval and data protection guidelines.
