# Supplementary material for: Genetic and non-genetic determinants of vitamin D status: a polygenic score analysis in elite athletes
Source: Front Genet. 2026 May 28;17:1838157. doi: 10.3389/fgene.2026.1838157 (PMC13252910; doi:10.3389/fgene.2026.1838157)
Supplement: Supplementary file 1 [file Table1.docx]

**Supplementary Table 1** Multiple linear regression predicting serum 25-hydroxyvitamin D levels

| **Variable** | **Estimate** | **95% CI** | ***p*-value** |
| --- | --- | --- | --- |
| (Intercept) | 72.12 | 67.72, 76.51 | < .001 * |
| PGS^‡^ | 4.04 | 1.65, 6.44 | < .001 * |
| Age^‡^ | 8.59 | 4.99, 12.19 | <. 001 * |
| cw-D-UVB^‡^ | 7.18 | 4.62, 9.74 | <. 001 * |
| Sex |  |  |  |
| *Male* | – | – |  |
| *Female* | -2.45 | -7.64, 2.74 | .354 |
| Competition environment |  |  |  |
| *Indoor* | – | – |  |
| *Outdoor* | 4.39 | -2.44, 11.22 | .207 |
| Supplementation |  |  |  |
| *No* | – | – |  |
| *Yes* | 20.86 | 8.06, 33.65 | .001 * |
| *Unknown* | -4.58 | -9.51, 0.35 | .069 |
| Genetic Ancestry^‡^ |  |  |  |
| *PC1* | 0.79 | -1.94, 3.52 | .57 |
| *PC2* | 2.64 | 0.57, 4.72 | .012 * |
| *PC3* | 1.59 | -0.92, 4.11 | .214 |
| *Note.* Multiple linear regression was calculated on the full dataset. Numeric variables (marked with ‡) were standardized. CI, confidence interval; cw-D-UVB, cumulative and weighted daily ambient UVB dose at wavelengths that can induce vitamin D synthesis; PGS, polygenic score; PC, principal component. Significant predictors (*p* < .05) are marked with an asterisk (*). | | | |

**Supplementary Table 2** Polygenic score (PGS) performance evaluation

|  | ***Full model*** | | ***Null model*** | | ***PGS*** | |
| --- | --- | --- | --- | --- | --- | --- |
| **Fold** | ***R*^2^** | **Adj. *R*^2^** | ***R*^2^** | **Adj. *R*^2^** | ***R*^2^** | **Adj. *R*^2^** |
| **1** | 0.15 | 0.04 | 0.15 | 0.05 | 0 | -0.01 |
| **2** | 0.28 | 0.2 | 0.28 | 0.2 | 0 | -0.01 |
| **3** | 0.3 | 0.22 | 0.29 | 0.21 | 0.01 | 0 |
| **4** | 0.47 | 0.4 | 0.45 | 0.39 | 0.02 | 0.02 |
| **5** | 0.39 | 0.32 | 0.39 | 0.32 | 0 | -0.01 |
|  |  |  |  |  |  |  |
| **Mean (SD)** | 0.32 (0.12) | 0.23 (0.14) | 0.31 (0.12) | 0.24 (0.13) | 0.01 (0.01) | 0 (0.01) |
|  |  |  |  |  |  |  |
| **Fold** | **MAE** | **RMSE** | **MAE** | **RMSE** | **MAE** | **RMSE** |
| **1** | 17.67 | 23.3 | 17.66 | 23.31 | -0.01 | 0.01 |
| **2** | 22.6 | 30.63 | 22.54 | 30.7 | -0.05 | 0.07 |
| **3** | 17.61 | 24.72 | 18.05 | 24.94 | 0.43 | 0.22 |
| **4** | 14.45 | 19.68 | 15.14 | 20.06 | 0.69 | 0.39 |
| **5** | 20.89 | 28.04 | 20.89 | 28.04 | 0 | 0 |
|  |  |  |  |  |  |  |
| **Mean (SD)** | 18.64 (3.18) | 25.27 (4.24) | 18.86 (2.9) | 25.41 (4.13) | 0.21 (0.33) | 0.14 (0.16) |
| *Note.* MAE, mean absolute error; PGS, polygenic score; RMSE, root mean squared error. | | | | | | |
